# Circular RNA DHX33 promotes malignant behavior in ccRCC by targeting miR-489-3p/MEK1 axis

**DOI:** 10.18632/aging.103550

**Published:** 2020-07-27

**Authors:** Jie Wang, Jian-Qiu Zhang, Xiao-Lei Zhao, Jing-Yu Lu, Ze-Ming Weng, Zhen-Min Ding, Feng-Qiang Yang

**Affiliations:** 1 Department of Urology, Ninghai First Hospital, Zhejiang 315600, China; 2 Department of Urology, Ninghai Hospital, Branch of Shanghai Tenth People’s Hospital, Zhejiang 315600, China; 3 Department of Urology, Huaihe Hospital of Henan University, Kaifeng 475000, China; 4 Department of Anesthesia, Shanghai East Hospital, Tongji University School of Medicine, Shanghai 200120, China; 5 Department of Urology, Shanghai Tenth People’s Hospital, Tongji University, Shanghai 200072, China

**Keywords:** clear cell renal cell carcinoma, circDHX33, miR-489-3p, MEK1

## Abstract

Mounting evidence indicates that circular RNAs modulate the initiation of clear cell renal cell carcinoma (ccRCC). However, their specific roles in the malignancy of ccRCC is understudied. Here, we present a novel circular RNA, circDHX33, that is up-regulated in ccRCC cell lines and tissues. Upregulated circDHX33 in ccRCC patients significantly correlates with advanced TNM stage and metastasis. Suppressing circDHX33 expression inhibits the proliferation and invasion of cultured cells, and suppresses tumor growth *in vivo*. Mechanistically, we show that circDHX33 promotes ccRCC progression by sponging miR-489-3p and modulating MEK1 expression. In conclusion, our findings suggest that circDHX33 plays a role in promoting ccRCC via the miR-489-3p/MEK1 axis and may serve as a novel therapeutic target for the treatment of ccRCC patients

## INTRODUCTION

Renal cell carcinoma (RCC) is a type of cancer affecting renal tubular epithelial cells, and has a high mortality rate [1]. It has many subtypes with clear cell renal cell carcinoma (ccRCC) being the most common type [2]. The mainstay treatment for ccRCC is surgery [3]. Although recent advances in its management have improved the survival rate of ccRCC, its prognosis following metastasis or recurrence remains poor [4, 5]. It is therefore necessary to improve our understanding on the molecular basis of this cancer.

Circular RNA (circRNA) is a novel class of endogenous RNA formed via covalent joining of the 3’ end to 5’ ends of RNA molecules [6, 7]. Mounting evidence suggests that circRNAs expression is dysregulated in various diseases, including cancers. Ge et al reported that low circMTO1 expression leads to low proliferation and invasion in colorectal cancer through a mechanism that involves the Wnt/beta-catenin signaling pathway [8]. Shao et al have reported that hsa_circ_0075341 promotes cervical cancer by sponging miR-149-5p [9]. Elsewhere, it was found the hsa_circ_0002577/miR-197/CTNND1 axis promotes Wnt/β-catenin signaling to promote endometrial carcinoma progression [10]. However, the mechanisms through which most circRNAs influence tumor progression remain unknown.

In this study, a novel 399 bp circRNA (hsa_circ_0000740), named circDHX33 is reported. This circRNA is formed by the splicing of *DHX33* mRNA. We find that circDHX33 is elevated in ccRCC patients and correlates with advanced metastasis and clinical stage. Mechanistically, circDHX33 promotes ccRCC by sponging miR-489-3p thereby upregulating MEK1 expression, accelerating cancer growth and metastasis. Collectively, these results imply that circDHX33/miR-489-3p/MEK1 axis could be therapeutically targeted to improve ccRCC outcomes.

## RESULTS

### circDHX33 is highly expressed in ccRCC

**Figure 1:**
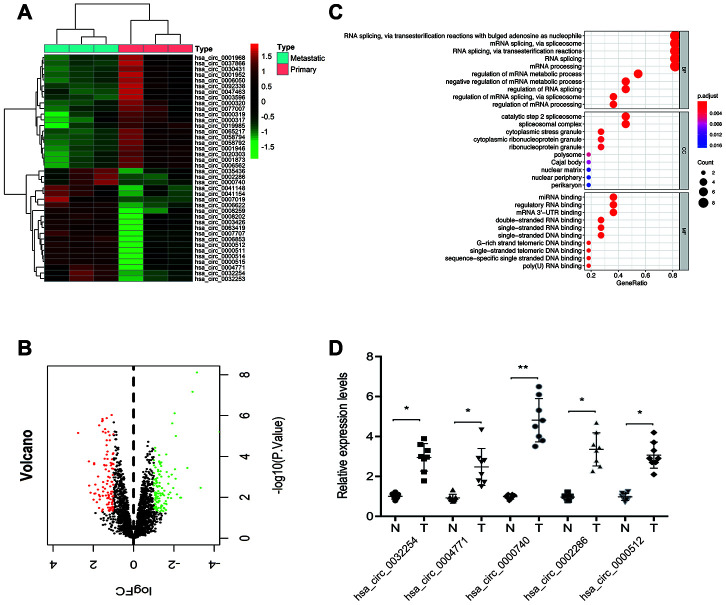
**Screening of ccRCC-related circRNAs in GSE137836 dataset.** (**A**, **B**) Hierarchical clustering analysis and volcano plots of significantly differentially expressed circRNAs in ccRCC tissues from the GSE137836 dataset. (**C**) GO enrichment analysis for dysregulated circRNAs gene symbols. (**D**) 5 upregulated circRNAs in 8 paired ccRCC tissues was determined by using RT-qPCR. *p<0.05, **p<0.01.

To detect aberrantly expressed circRNAs in ccRCC tissues, we took advantage of a microarray expression profile data of circRNAs in ccRCC tissues (GSE137836). Analysis revealed that 211 circRNAs were differentially expressed between metastatic and primary groups (fold change >2.0) and *p* < 0.05 (Figure 1A, 1B). GO term analysis revealed that these differentially expressed circRNAs are involved in various physiological processes, molecular functions, and cell signaling pathways (Figure 1C). Next, we selected the top 5 upregulated circRNAs in dataset GSE137836 and used RT-qPCR to validate their upregulation in ccRCC tissues. A novel circRNA hsa_circ_0000740 (circDHX33) was found to be the most significantly upregulated circRNA in the ccRCC tissues relative to normal controls (Figure 1D).

**Figure 2:**
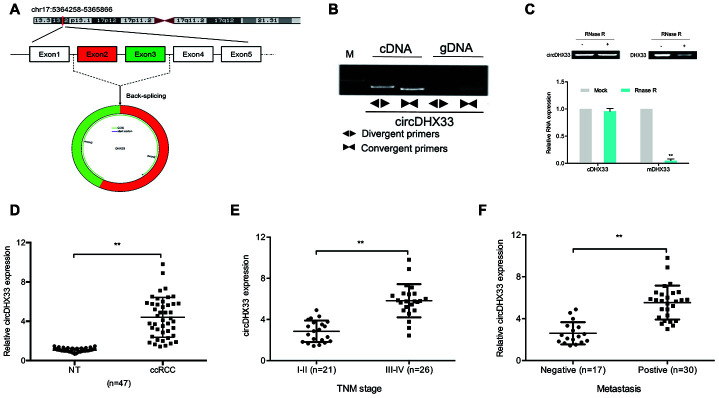
**circDHX33 was highly expressed in ccRCC.** (**A**) Schematic diagram of circDHX33. (**B**) Random hexamer or oligo (dT)18 primers were used in the reverse transcription experiments. (**C**) The relative RNA levels were determined by qRT-PCR. (**D**) The expression of circDHX33 in 47 paired ccRCC tissues was detected by qRT-PCR. (**E**, **F**) High circDHX33 expression was linked to advanced TNM stage and metastasis in ccRCC patients. *p<0.05, **p<0.01.

We therefore decided to further characterize circDHX33. CircDHX33, which is located on chromosome 17, consists of 2 exons (Exons 2-3) that are spliced from DHX33 mRNA (Figure 2A). We then confirmed the circular characteristics of circDHX33. To this end, genomic DNA and cDNA from some ccRCC tissue were used as templates to amplify circDHX33 using divergent primers. We did not obtain amplification products for genomic DNA (Figure 2B). Moreover, our data revealed that circDHX33 resisted RNase R, revealing that circDHX33 circularized the RNA (Figure 2C). RT-qPCR showed that circDHX33 expression in ccRCC was significantly enhanced and correlated with advanced TNM stage and metastasis (Figure 2D–2F). These data indicate that circDHX33 has an important function in ccRCC progression.

### Knockdown of circDHX33 inhibits the proliferation and invasion of ccRCC cells

**Figure 3:**
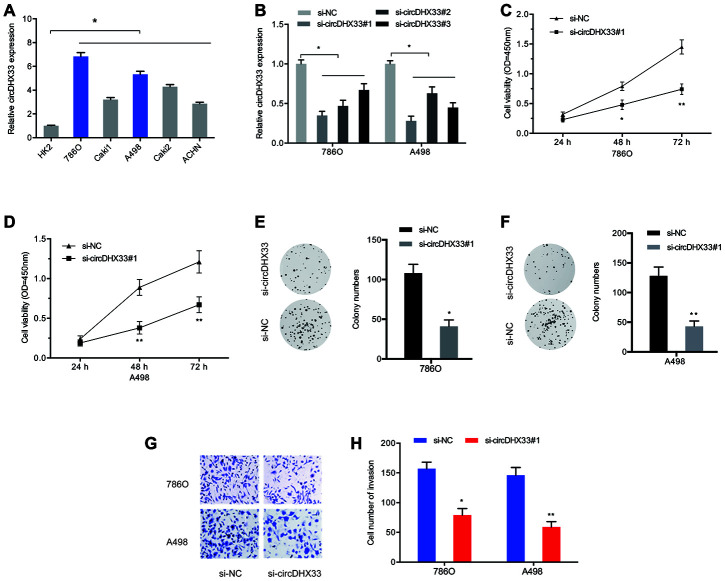
**circDHX33 promoted the proliferation and invasion in ccRCC cells.** (**A**) The expression of circDHX33 in RCC cell lines. (**B**) The efficiency of circDHX33 knockdown in 786O and A498 cells. (**C**–**F**) Cell viabilities were assessed by CCK-8 and colony formation and tests. (**G**, **H**) Cell invasion was assess using Transwell assay. *p<0.05, **p<0.01.

To assess the biological functions of circDHX33 in ccRCC progression, mRNA levels of circDHX33 in RCC cells were estimated. Results indicated that circDHX33 level was markedly upregulated in the RCC cell lines 786O, Caki1, A498, Caki2, and ACHN, relative to HK2 cell lines (Figure 3A). We then transfected si-circDHX33 into 786O and A498 cell lines (Figure 3B). Cell proliferation analysis using CCK-8 and colony formation tests indicated that circDHX33 inhibition strongly decreased proliferation (Figure 3C–3F). Analysis of cell invasion showed that down-regulation of circDHX33 markedly inhibited invasion of 786O and A498 cells (Figure 3G–3H). These findings suggested that circDHX33 silencing represses the proliferation and metastasis of RCC cells in vitro.

### circDHX33 targets miR-489-3p as a miRNA sponge

**Figure 4:**
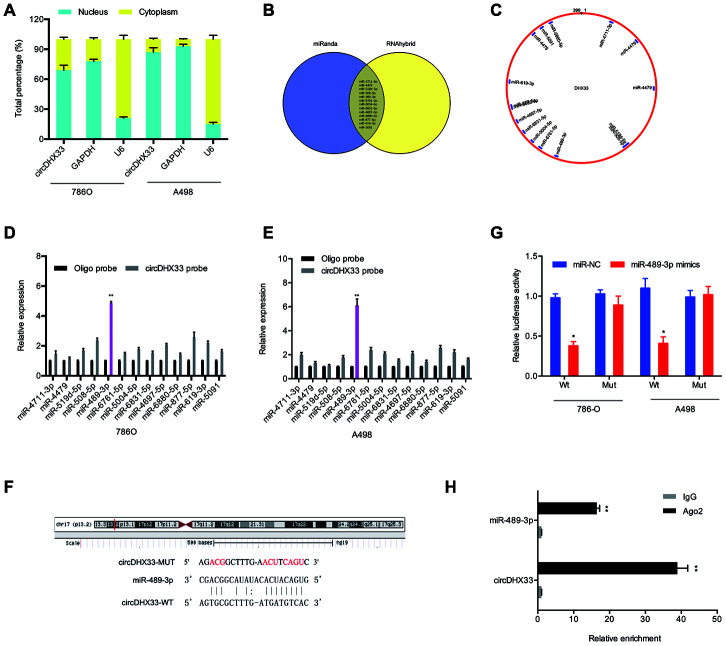
**circDHX33 targeted miR-489-3p as a miRNA sponge.** (**A**) circDHX33 distribution was explored by subcellular fractionation assay. (**B**, **C**) Target miRNAs of circDHX33 was predicted by miRanda and RNAhybrid. (**D**, **E**) The relative levels of miRNA candidates in 786O and A498 cells lysates were detected by pull-down assay. (**F**) MiR-489-3p had the complementary sites with the circDHX33. (**G**) MiR-489-3p mimics reduced the luciferase activity of the circDHX33-WT group. (**H**) The correlation between circDHX33 and miR-489-3p was confirmed by RIP assay. *p<0.05, **p<0.01.

Multiple studies have demonstrated that circRNAs modulate cellular function by sponging miRNA and therefore suppressing their function [11–13]. The nuclear-cytoplasmic fractionation assay showed that the cytoplasmic fraction contained higher circDHX33 levels relative to the nuclear fraction (Figure 4A). We then used miRanda and RNAhybrid databases to predict potential target miRNAs for circDHX33 (Figure 4B and 4C). Pull-down assay revealed that miR-489-3p was strongly associated with the circDHX33 probe in both 786-O and A498 cells (Figure 4D-4E). Figure 4F showed a schematic representation of miR-489-3p binding sites in circDHX33. A luciferase assay revealed that the miR-489-3p mimics suppressed luciferase activity in circDHX33-WT group (Figure 4G). The RIP assay confirmed an association between circDHX33 and miR-489-3p in RCC cells (Figure 4H).

**Figure 5:**
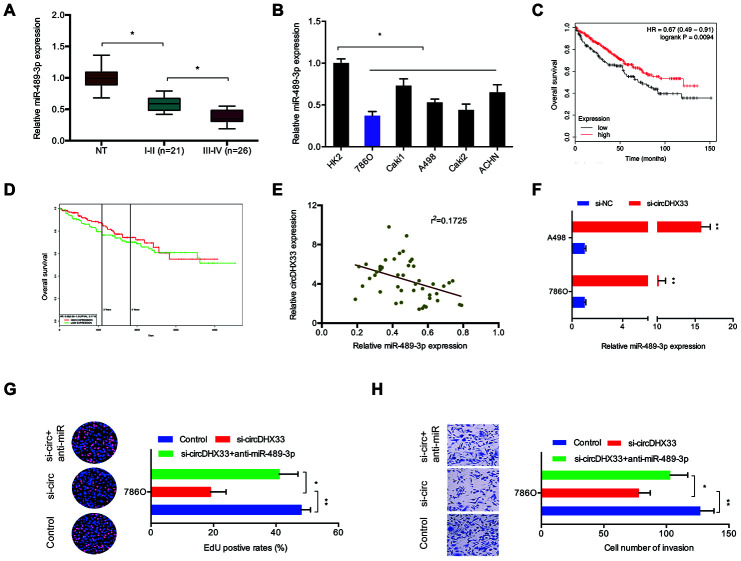
**circDHX33/miR-489-3p axis in ccRCC.** (**A**, **B**) The level of miR-489-3p in ccRCC tissues and cell lines was estimated with RT-qPCR. (**C**, **D**) Low miR-489-3p levels correlated with poor overall survival rate in patients. (**E**) circDHX33 expression was negatively correlated with miR-489-3p expression in ccRCC tissues. (**F**) circDHX33 negatively regulated expression of miR-489-3p in 786O and A498 cells. (**G**, **H**) miR-489-3p suppression abolished the effects of circDHX33 inhibition on 786O cells proliferation and invasion abilities. *p<0.05, **p<0.01.

We next explored miR-489-3p expression in ccRCC using RT-qPCR analysis and discovered that it was downregulated in ccRCC tissues and cell lines (Figure 5A, 5B). Kaplan-Meier analysis indicated that low miR-489-3p levels correlated with poor RCC survival (Figure 5C, 5D). Further analysis showed that circDHX33 expression was negatively correlated with miR-489-3p level in ccRCC tissues (Figure 5E). CircDHX33 suppression elevated miR-489-3p expression in 786O cells and A498 cells (Figure 5F). Moreover, rescue experiments indicated that miR-489-3p inhibitors abolished the effects of circDHX33 silencing on786O cells proliferation and invasion abilities (Figure 5G, 5H). Together, these findings suggest that circDHX33 sponges miR-489-3p in ccRCC.

### MEK1 is a downstream target of miR-489-3p in ccRCC

**Figure 6:**
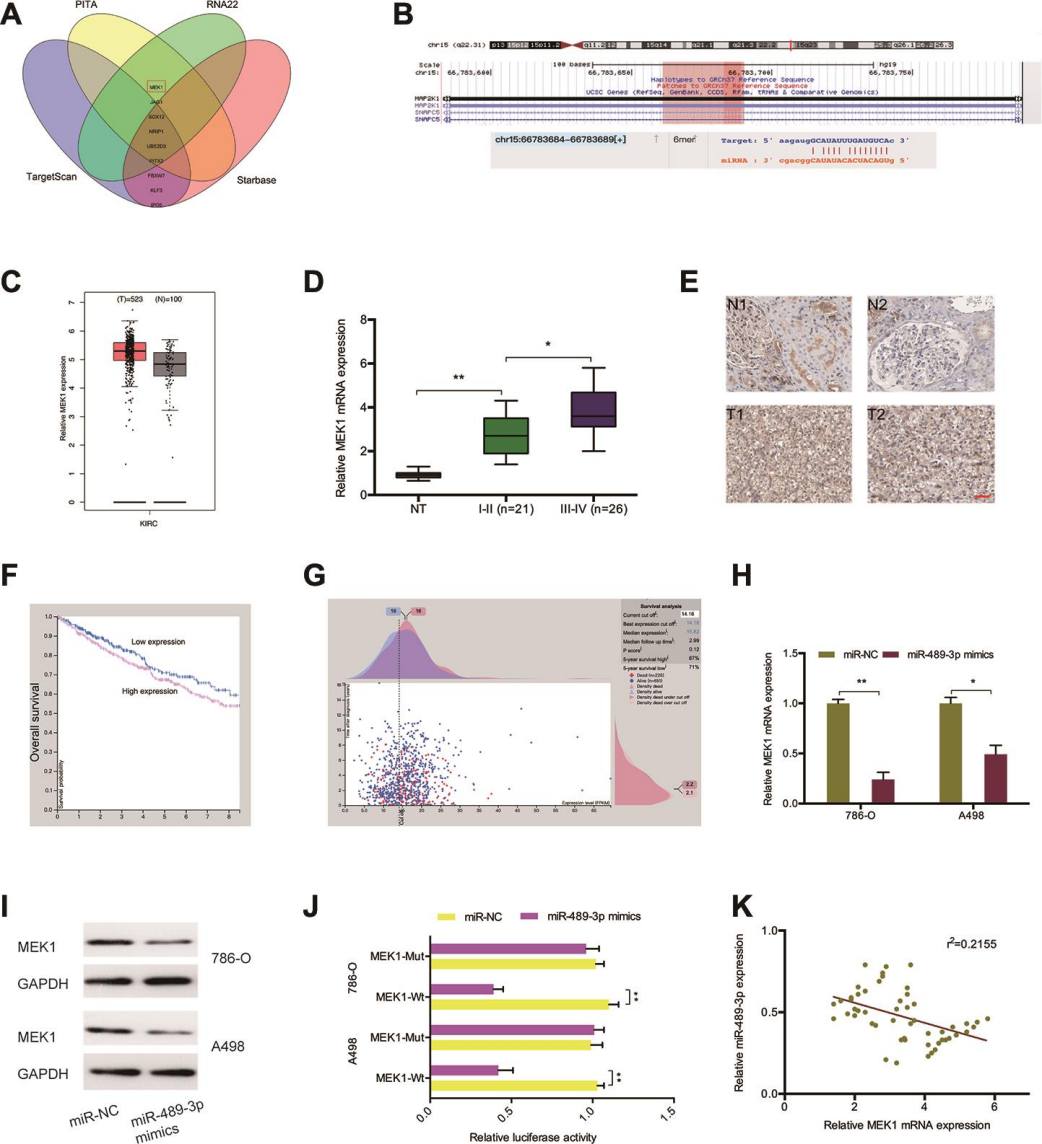
**MEK1 served as the target of miR-489-3p.** (**A**, **B**) MEK1 was a potential putative target gene of miR-489-3p. (**C**) MEK1 expression was significantly upregulated in KIRC tissues. (**D**, **E**) MEK1 expression in ccRCC tissues was determined by RT-qPCR and IHC. (**F**, **G**) High MEK1 expression was significantly associated with poor prognosis in patients. (**H**, **I**) MiR-489-3p mimics reduced MEK1 expression in 786O and A498 cells. (**J**) MiR-489-3p mimics reduced luciferase activity of the MEK1-Wt group. (**K**) miR-489-3p expression was negatively correlated with MEK1 expression in ccRCC tissues. KIRC: Kidney renal clear cell carcinoma. *p<0.05, **p<0.01.

To explore the mechanism by which miR-489-3p functions in RCC, we searched TargetScan, Starbase, PITA, and RNA22 with the aim of identifying its target genes. This analysis revealed MEK1 as a target of miR-489-3p with a specific binding site at the 3’-UTR (Figure 6A, 6B). GEPIA analysis showed that MEK1 expression was upregulated in ccRCC tissues (Figure 6C), and this was validated by RT-qPCR and IHC analysis (Figure 6D, 6E). A Kaplan-Meier analysis indicated that elevated MEK1 expression significantly correlated with poor prognosis (Figure 6F, 6G). MiR-489-3p mimics significantly suppressed MEK1 expression in 786O and A498 cells (Figure 6H, 6I). Additionally, a luciferase reporter assay revealed that miR-489-3p mimics significantly suppressed luciferase activity in the MEK1-Wt group but not in the MEK1-Mut group (Figure 6J). Moreover, correlation analysis showed that miR-489-3p expression negatively correlated with MEK1 expression in ccRCC tissues (Figure 6K). Taken together, these findings indicate that MEK1 is downstream target of miR-489-3p in ccRCC.

### circDHX33 promotes ccRCC progression through the miR-489-3p/MEK1 axis

**Figure 7:**
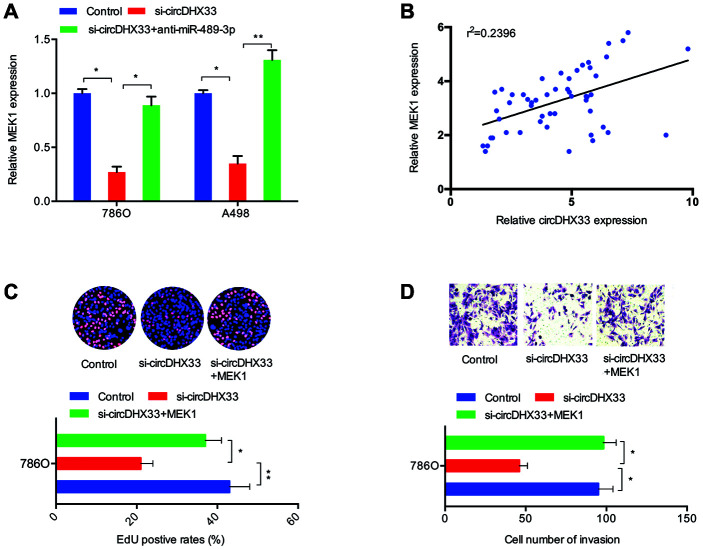
**circDHX33/miR-489-3p/MEK1 axis in ccRCC.** (**A**) MiR-489-3p suppression in 786O and A498 cells abolished the effects of circDHX33 inhibition on MEK1 expression. (**B**) circDHX33 expression was positively correlated with MEK1 expression in ccRCC tissues. (**C**, **D**) MEK1 overexpression abolished the effects of circDHX33 inhibition on 786O cells proliferation and invasion abilities. *p<0.05, **p<0.01.

Next, we investigated whether circDHX33 influences ccRCC progression via miR-489-3p/MEK1 axis. RT-qPCR showed that silencing circDHX33 decreased MEK1 expression in 786O cells and A498 cells, and that this effect was reversed by inhibitors of miR-489-3p (Figure 7A). Correlation analysis revealed a positive association between circDHX33 expression and MEK1 expression in ccRCC tissues (Figure 7B). Finally, EdU and transwell assays showed that MEK1 overexpression abolished the effects of circDHX33 inhibition on the proliferation and invasion in 786O cells (Figure 7C, 7D).

### circDHX33 promotes ccRCC cell growth *in vivo*

**Figure 8:**
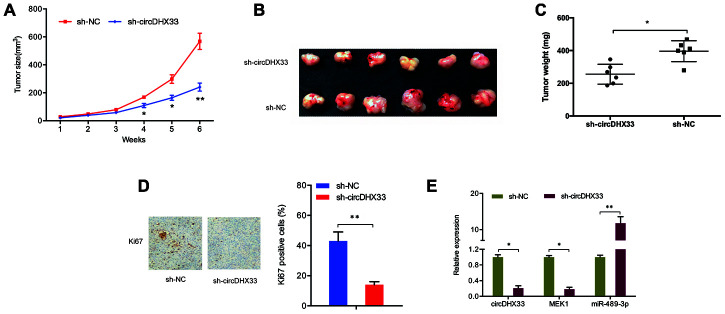
**circDHX33 promoted tumor growth in vivo.** (**A**–**C**) circDHX33 knockdown reduced tumor growth in vivo. (**D**) circDHX33 knockdown reduced Ki67 expression in mice tissues. (**E**) circDHX33 knockdown reduced MEK1 levels and increased miR-489-3p levels in mice tissues. *p<0.05, **p<0.01.

Next, we determined the functions of circDHX33 in tumor growth *in vivo*. A significant inhibition of tumor growth was observed following circDHX33 knockdown (Figure 8A). Measurement of tumor volume and weight indicated they were markedly lower in the circDHX33 knockdown group relative to control group (Figure 8B, 8C). IHC analysis of Ki67 staining revealed that circDHX33 knockdown suppressed tumor cell proliferation *in vivo* (Figure 8D). RT-qPCR assay revealed that miR-489-3p was upregulated in sh-circDHX33 group and MEK1 was downregulated in the sh-circDHX33 group (Figure 8E). Taken together, these observations indicate that circDHX33 may enhance MEK1 expression by acting as a ceRNA for miR-489-3p, thereby promoting ccRCC progression.

## DISCUSSION

The initiation, development and progression of ccRCC are complex processes that involve a wide range of internal and external factors [14, 15]. CircRNAs have been reported to play important roles in the pathogenesis of ccRCC [16]. Huang et al showed that elevated levels of circRNA ABCB10 promoted the progression of cancer and predicted poor prognosis [17]. Xue et al found that circ-AKT3 reduced ccRCC metastasis by modulating the miR-296-3p/E-cadherin axis [18]. Another study reported that circPCNXL2 promoted the invasiveness and proliferation of ccRCC cells by regulating the miR-153/ZEB2 axis [19]. However, the mechanisms by which circRNAs influence ccRCC are largely unknown.

In this study, we uncovered a novel circRNA (hsa_circ_0000740/circDHX33). circDHX33 if formed by DHX33 RNA splicing and comprises a circular fragment of exon 2 and 3. DHX33 is a member of the DEAD/DEAH box family of RNA helicases involved in cancer progression [20]. Here, we find that circDHX33 is markedly upregulated in ccRCC cell lines and tissues. CircDHX33 suppression decreased the proliferative capacity and invasiveness of ccRCC cells *in vitro* and suppressed the growth of tumors *in vivo* suggesting circDHX33 is a novel therapeutic target for ccRCC. There is evidence that circRNAs modulates miRNA function by acting as a miRNA sponge [12]. MiR-489-3p is a tumor suppressor in many types of cancers. Inhibition of miR-489-3p enhanced metastasis of osteosarcoma by stimulating PAX3-MET axis [21]. It was established that miR-489 inhibited the proliferative capacity and invasiveness of bladder cancer cells [22]. LINC01446 promoted the progression of glioblastoma cells by modulating miR-489-3p/TPT1 axis [23]. Herein, we uncover that circDHX33 binds to miR-489-3p in ccRCC. We also show that MiR-489-3p is reduced in ccRCC. Moreover, we find that circDHX33-mediated suppression of cell proliferation and invasion are mitigated by miR-489-3p inhibitors. Together, these data suggest that circDHX33 exerts oncogenic effects by sponging miR-489-3p in ccRCC.

Mitogen-activated protein kinase (MEK) plays critical roles in tumor progression [24]. Huang et al reported that miR-101 regulates proliferative capacity and apoptosis in diffuse large B-cell lymphoma through MEK1 and ERK/MAPK pathway [25]. Zhang et al demonstrated that circDLST promotes gastric cancer progression by modulating the miR-502-5p/NRAS/MEK1/ERK1/2 axis [26]. In this study, we report that MEK1 is upregulated and positively correlates with circDHX33 expression. We find that MEK1 is directly targeted by miR-489-3p and circDHX33 indirectly regulates MEK1 expression. Moreover, the effects of circDHX33 inhibition on cell proliferation and invasion were reversed by MEK1 overexpression, suggesting MEK1 might be important in ccRCC development. These findings indicate that circDHX33 might regulate ccRCC growth and metastasis by regulating the miR-489-3p/MEK1 axis.

## CONCLUSION

This is the pioneer study showing that circDHX33 has an oncogenic role ccRCC both *in vivo and in vitro*. Mechanistically, we show that the effects of circDHX33 are mediated via the circDHX33/miR-489-3p/MEK1 axis. circDHX33 might therefore be an ideal therapeutic target for ccRCC treatment.

## MATERIALS AND METHODS

### Clinical samples

We collected 47 paired ccRCC tissues and adjacent normal tissues from patients at the department of urology, Ninghai First Hospital and Huaihe Hospital-Henan University. The diagnosis of ccRCC was confirmed by 2 independent pathologists. Ethical approval for this study was granted by the ethics committee of the Ninghai First Hospital and Huaihe Hospital of Henan University. All participants provided written informed consents for sample collection.

### Cell culture and treatment

Renal cancer cell lines, ACHN, Caki2, A498, Caki1, and 786O, and normal kidney epithelial cells line (HK2) were obtained from the American type culture collection (ATCC). These cells were grown in Dulbecco’s modified Eagle’s medium (DMEM; Gibco, Carlsbad, CA, USA) containing 1% penicillin/streptomycin (pen/strep) (Invitrogen, Carlsbad, CA, USA) and with 10% FBS (Gibco) in a humidified incubator, at 37ºC, 5% CO_2_.

circDHX33 silencing-specific siRNAs (si-circDHX33#1 sequence is 5′- AGATCCACCAGGTTGGCTATA-3′; si-circDHX33#2 sequence is 5′- ATCCACCAGGTTGGCTATACA-3′; si-circDHX33#3 sequence is 5′- CAGATCCACCAGGTTGGCTAT-3′) were purchased from GenePharma (Shanghai, China). MiR-489-3p inhibitors and mimics, pcDNA.3.1 MEK1 recombinant plasmid and the respective control RNAs were purchased from Ribobio (Guangzhou, China) siRNA transfections were done with lipofectamine 2000 (Invitrogen) following the manufacturer’s directions.

### Cell proliferation assay

Cell Counting Kit-8 (CCK-8; Roche, Basel, Switzerland) was used to assess the proliferation of RCC cells. After seeding 1×10^4^ transfected cells in 96-well plates, they were cultured for 24, 48 or 72 hours. 10μl of CCK-8 was then added to the cells and cultured for 2 hours. The absorbance of the plate was read at 450nm using a microplate reader.

### Colony formation assay

To assess the ability of cells to form colonies, 1×10^3^ cells were cultured in 6-well plates with DMEM medium for 2 weeks. The culture medium was replaced at intervals of 3 days. Next, 1X PBS was used to wash colonies, which were then fixed with methanol for staining with crystal violet after 2 weeks in culture. The number of colonies were counted manually.

### Transwell invasion assay

We evaluated cell invasion using transwell chambers. Briefly, tranwell chambers were coated with 80μl of Matrigel and 3x10^5^ cells in 400μl of serum-free DMEM seeded onto the upper chamber. The lower chamber was filled with 400μl DMEM supplemented with 10% FBS. 24 hours later, un-migrated cells were scrapped off with a cotton swab while invasive cells were stained with 0.1% crystal violet before counting under a microscope.

### RNA extraction and quantitative real-time PCR (RT-qPCR)

Cells and tissues were lysed with Trizol reagent (Invitrogen) to isolate total RNA. RT-qPCR was performed using First Strand cDNA Synthesis Kit (TakaRa, China) as per the manufacture’s protocol. RT-qPCR analysis was done using the ChamQ SYBR qPCR Master Mix (Vazyme Biotech, Nanjing, China) on a Roche LC 96 qPCR system (Roche, Germany). Relative mRNA levels were estimated using the 2^-ΔΔCT^ method. Primer sequences were as follows: circDHX33, 5′-GCTTGTCTCCGTCTTCCAGA-3′ (forward) and 5′- CTGAAATTGCTTCACGCAGA-3′ (reverse); miR-489-3p, 5′- GGGGTGACATCACATATAC-3′ (forward) and 5′-CAGTGCGTGTCGTGGAGT-3′ (reverse); MEK1, 5′- CAAGAAGAAGCCGACGCCCAT-3′ (forward) and 5′- GACGCCAGCAGCATGGGTTG-3′; U6, 5'- CGCGCTTCGGCAGCACATATACT-3' (forward) and U6, 5'- ACGCTTCACGAATTTGCGTGTC-3' (reverse); GAPDH, 5'-GAGTCAACGGATTTGGTCGT-3' (forward) and 5'-TTGATTTTGGAGGGATCTCG-3' (reverse).

### Dual-luciferase reporter assay

circDHX33 segment or MEK1 3’UTR was inserted into pGL3-control plasmid. Mutations on circDHX33 and the MEK1 3’UTR were induced by changing the conserved binding sites of miR-489-3p using a gene mutation kit (Takara). The mutated sequence or wild-type seed region were co-transfected with miR-489-3p mimics or miR-NC into RCC cells using Lipofectamine 2000. Luciferase activity was measured using the Dual-Luciferase® reporter assay system (Promega, Madison, WI, USA) according to the manufacturer’s protocol.

### RNA immunoprecipitation (RIP)

RIP was done using the Magna RIP Kit (Millipore, Billerica, MA, USA) as per the manufacturer’s instructions. Antibodies against argonaute2 (anti-AGO2) and immunoglobulin G (IgG) were used for the RIP assays. Purified RNAs were extracted and the enrichment of circDHX33 and miR-489-3p analyzed by RT-qPCR, as described previously [11].

### Tumorigenesis assay

Twelve (12) female nude mice (athymic BALB/c) aged 4-6 weeks and weighing 16-20g were housed in pathogen-free conditions. The animals were randomly split into two groups (the sh-NC and sh-circDHX33 groups). 200μL of 1×10^7^ cells were subcutaneously injected into the back flanks of the animals. Tumors was measured each week after the tumor became visible and tumor volume calculated following with the formula: volume = (0.5 × length × width^2^). After 6 weeks following implantation, the mice were sacrificed and tumors were excised, weighed and photographed.

### Bioinformatics analysis

Survival analysis for RCC patients with miR-489-3p expression was performed using the KM plot database (http://www.kmplot.com) and PROGgeneV2 (http://genomics.jefferson.edu/proggene/results.php). The expression of MEK1 in RCC was determined by GEPIA (gepia.cancer-pku.cn). PROTEIN ATLAS (https://www.proteinatlas.org/) was used to investigate the prognosis of MEK1 in RCC. The circDHX33 specific binding with miRNAs was identified by using miRanda and RNAhybrid.

### Statistical analysis

All data are presented as mean ± standard deviation (SD). Statistical analysis was done using SPSS 20.0 (SPSS, Chicago, IL, USA). Student’s t-test and one-way ANOVA tests were utilized to compare differences between groups, respectively. *p* < 0.05 was considered statistically significant.
